# Enhancing the Prediction of Emotionally Intelligent Behavior: The PAT Integrated Framework Involving Trait EI, Ability EI, and Emotion Information Processing

**DOI:** 10.3389/fpsyg.2018.01078

**Published:** 2018-07-02

**Authors:** Ashley Vesely Maillefer, Shagini Udayar, Marina Fiori

**Affiliations:** ^1^Department of Organizational Behavior, University of Lausanne, Lausanne, Switzerland; ^2^Swiss National Centre of Competence in Research LIVES, University of Lausanne, Lausanne, Switzerland

**Keywords:** emotional intelligence, trait EI, ability EI, emotion information processing, integrated framework

## Abstract

Emotional Intelligence (EI) has been conceptualized in the literature either as a dispositional tendency, in line with a personality trait (trait EI; Petrides and Furnham, 2001), or as an ability, moderately correlated with general intelligence (ability EI; Mayer and Salovey, 1997). Surprisingly, there have been few empirical attempts conceptualizing how the different EI approaches should be related to each other. However, understanding how the different approaches of EI may be interwoven and/or complementary is of primary importance for clarifying the conceptualization of EI and organizing the literature around it. We introduce a theoretical framework explaining how trait EI, ability EI, and emotion information processing – a novel component related to EI recently introduced in the literature (e.g., Fiori and Vesely Maillefer, 2018) – may contribute to effective emotion-related performance and provide initial evidence supporting its usefulness in predicting EI-related outcomes. More specifically, we show that performance in a task in which participants had to infer the mental and emotional states of others, namely a Theory of Mind task, was predicted jointly (e.g., interaction effects) by trait EI, ability EI, and emotion information processing, after controlling for personality and IQ (*N* = 323). Our results argue for the importance of investigating the joint contribution of different aspects of EI in explaining variability in emotionally laden outcomes.

## Introduction

For the past almost three decades, there has been debate surrounding the definition and measurement of Emotional Intelligence (EI; Matthews et al., 2002; Zeidner et al., 2008, 2012). The dominant theoretical frameworks remain *ability* EI and *trait EI.* The *ability EI* (AEI) approach conceptualizes EI as an ability, framing it as a form of intelligence, specifying that cognitive processing is implicated in emotions, and that it should thus be assessed through performance measures (Mayer and Salovey, 1997; Freeland et al., 2008). It was formulated from the four-factor Salovey-Mayer model (Mayer and Salovey, 1997), is now being refined upon (MacCann and Roberts, 2008), and a three-factor solution has been acknowledged (removal of ‘using emotions’) as a better empirical fit (e.g., Keele and Bell, 2008; Fiori et al., 2014). The general trait EI approach is related to personality and most often focuses on the emotional self-efficacy of the individual that is measured through self-report scales (Palmer and Stough, 2001; Petrides and Furnham, 2001; Schutte et al., 2009). *Trait EI* (TEI) has been researched extensively by scholars such as Petrides and Furnham (2001) and many others, who have developed a range of trait models (e.g., Palmer and Stough, 2001; Wong et al., 2007). Some research has also utilized “mixed models” of EI (e.g., Bar-On, 2006), which include a combination of personality traits, dispositions, and competencies related to emotion, though these have shown to have little incremental variance when predicting important outcomes (Joseph et al., 2015).

Establishing the criteria necessary for the accurate conceptualization of EI has involved many challenges. More current discussion on EI has introduced the idea of *emotion information processing* (EIP) as an additional component related to EI that may account for variability in emotionally intelligent behavior (Fiori and Vesely Maillefer, 2018). The way individuals process emotion information, such as how they acquire, perceive, encode, pay attention to, retain, and retrieve emotion information (Suedfeld and Tetlock, 1977), is an aspect that is little explored in the literature with respect to EI and that would capture how individuals experience emotions. Indeed, it is argued that EI also requires a fluid, experiential component able to differentiate individuals with real practical emotional skills from ‘intelligent machines’ that would be able to perform well on ability EI tests based solely on algorithmic, rather than emotional, processes (Ortony et al., 2007). The same concern emerges when observing individuals who may lack practical interactive skills, such as individuals with Asperger’s Syndrome, who may improve on ability EI tests through learning without being able to change their emotionally intelligent behavior in person (Montgomery et al., 2010). This is congruent with the idea that ability EI measures tap more into the knowledge people have about emotions rather than the application of it to real life situations (Fiori, 2009; Fiori and Vesely Maillefer, 2018).

The notion that emotion information processing is associated with EI is supported by a few studies that have investigated this issue. Individuals higher in TEI showed attentional biases toward positive, rather than neutral and negative, emotional stimuli (Lea et al., 2018). Further, a recent systematic review on EI and its relationship to both emotionally laden (hot) and not emotionally laden (cool) cognitive processes measured by computer lab tasks (go-nogo, IGT, stroop etc.) showed differential relationships between emotion information processing and different means of measuring EI (self-report ability, performance-based ability, and self-report mixed models; Gutiérrez-Cobo et al., 2016), with higher predictive value of performance-based ability on emotion information processing. The introduction of a separate but related concept, emotion attention regulation (EAR), which involves focusing emotion-related attention for the purpose of information processing rather than in order to regulate one’s own internal state (Elfenbein et al., 2017), has further reinforced the account that emotion information processing is an important component of the construct of EI.

Whether it should be represented as a trait or an ability, assessed using self-report or performance measures, or whether the constructs to date effectively describe EI in its practical application, what all of these theoretical frameworks share in common is their conceptualization of EI as a distinct construct from traditional IQ and personality, which facilitates the potential for prediction of, and influence on, various real-life outcomes (e.g., Ciarrochi et al., 2000; Petrides et al., 2007).

Surprisingly, there is little understanding regarding whether and how the different EI perspectives relate to one another. Furthermore, though some theoretical articles do discuss how trait EI and ability EI could jointly predict outcomes (e.g., Seal and Andrews-Brown, 2010), very few empirical contributions address the possibility of statistical interaction of approaches (for an exception see Salguero et al., 2015). And yet, understanding how the different attributes of the same underlying EI construct may be interwoven and/or complementary is of primary importance for clarifying the conceptualization of EI and organizing the literature around it. An attempt to theoretically understand different components of EI was made with the tripartite model (Mikolajczak, 2009; Mikolajczak et al., 2009), where three levels of EI, namely (1) knowledge – reflecting what people know about emotions, (2) ability – to apply this knowledge in real-world situations, and (3) traits – reflecting the propensity to behave in a certain way in emotional situations (typical behavior), are theoretically proposed. Although this conceptualization of the EI components is helpful in understanding the complexity of emotionally intelligent behavior, it does not provide specific directions regarding how such components would interact with each other in order to yield better performance. A comprehensive and developed model is the one proposed by Seal and Andrews-Brown (2010), which explains how different components of EI may interact predicting emotional intelligent behavior by means of a moderated-mediation framework. The authors identified three paradigms composing their integrative model of EI: emotional quotient that they viewed as preferred patterns of behavior, emotional ability that they considered as the potential capacity of the individual, and emotional competence, which represents the actual behaviors impacting performance. In the proposed model, the effect of trait EI on performance outcomes is mediated by emotional competence and this relationship is moderated by ability EI.

Our conceptualization of how different components of EI may interact to produce emotionally intelligent behavior presents similarities with the above-mentioned model, although we consider the different EI components on the same level. Specifically, we argue that each EI conceptualization represents a different attribute of EI, each of which interactively impact associated outcomes. In addition, we do not conceptualize emotional competence as the mechanism through which the other EI components may lead to higher performance; instead, relying on the vocational behavior literature (Bloom, 1976; Bellier, 2004) we consider emotional competence as the shared variance among the different EI components. Importantly, we introduce a new third approach among the different EI components, together with trait and ability EI–emotion information processing–and then provide initial evidence regarding the extent to which this novel framework predicts adaptive performance. Prior to introducing our study, we look to some theoretical and empirical reasoning in favor of integrating a combination of approaches for the purpose of predicting adaptive outcomes.

### Predictors of Emotionally Intelligent Performance

With this objective in mind, we turn to a discussion on the way in which the different EI components may contribute to effective performance. Inconsistencies in language and terminology have contributed to blurring the lines among the various EI definitions. Terms such as ability, trait, competence, capacity, and self-efficacy, are often utilized inconsistently, requiring us to query whether EI refers to the *potential* to behave in an emotionally intelligent manner, the *frequency* with which we do so on a daily basis (typical behavior), or the way we would behave if we were being evaluated at our best (maximal performance). Furthermore, when looking at emotionally intelligent behavior, the literature has also confused what we see as the outcome of having high EI versus the components of EI that account for these outcomes. For instance, some studies argue that having the ability to manage emotions as measured by the MSCEIT (an emotion knowledge based test) is equivalent to being able to regulate emotions in a specific situation, which is not necessarily the case (see Peña-Sarrionandia et al., 2015 for how the two constructs can be conceptualized). For instance, one may be able to employ a breathing technique during practice as well as understand conceptually that employing it would help to calm oneself down if upset, however, one may not be able to implement this technique in the ‘heat of the moment.’ Further, the situation may have an impact on whether one is able to reflect enough in order to call on the effective technique.

This difficult distinction between predictors and outcomes, and more specifically, ability versus performance, has also been discussed in the context of general intelligence and personality research (Chamorro-Premuzic and Furnham, 2004). The authors make the distinction between actual intellectual ability (including crystallized and fluid intelligence), and performance on IQ tests, though in recognition that the latter is a very strong predictor of the former. Their model then specifies also that certain personality factors have an impact on both actual ability, IQ test performance, and subjectively assessed intelligence, the latter of which also impacts IQ test performance (Chamorro-Premuzic and Furnham, 2004), emphasizing both the multiplicity of factors as well as their complex interrelationships that can all contribute to performance. For the purpose of clarity, emotionally intelligent behavior is here considered the general outcome with different EI components defining the individual components that aim to predict this outcome.

Looking broadly at general competence models also provides support for the integration of a range of attributes (traits, abilities, and processing) into a single model that should predict positive performance in a specific domain. An influential typology, known as Bloom’s Taxonomy utilized to assess performance in educational settings, is comprised of a variety of factors called KSAs: knowledge, skills, attitudes (Bloom et al., 1971; Bloom, 1976). Within this literature, work competence is based on these three cognitive domains: mental (Knowledge), affective (growth in feelings or emotional areas; Attitudes), and psychomotor (manual or physical skills; Skills), each of which jointly contributes to learning outcomes. Further, Kanfer and Ackerman (2005) discuss an overview of work competence that includes abilities, knowledge and skills, motivation, personality, and self- concept (such as self-confidence and self-efficacy) and indicate that work-related behavior can be predicted by both ability and non-ability characteristics. Their conceptualization tends to be focused on maximal performance (what the individual can do at their best) and also makes a distinction between competence and performance indicating that the latter is influenced by external (e.g., failed equipment) and transitory (e.g., temporary distraction) factors in addition to these internal factors such as traits, abilities, and skills (Kanfer and Ackerman, 2005).

A model known especially in the vocational behavior literature seems more comprehensive and describes professional competence as being comprised of three major components: savoir (declarative knowledge or having theoretical understanding of information), savoir-être (general attitude or tendency toward behavior of knowledge; behavioral competencies), and savoir-faire (the application of this knowledge or procedural knowledge; functional competencies). Each of these can predict performance outcomes independently as well as interact with each of its counterparts to create competence – the interaction of all three components (Bellier, 2004). The model is set within its specific context, which may also affect the outcome. This model is akin to the independent and interactive contribution of knowledge, application of such knowledge, and basic trait dispositions that may drive effective performance with all three required to reach ‘full competence,’ the most comprehensive predictor of performance.

Bearing on these theorizations, we propose a comprehensive and integrated approach in which we employ the above-mentioned components plus the new dimension of emotion information processing–representing how individuals react to contextual emotional stimuli– to predict ‘emotionally intelligent’ or ‘adaptive’ behavior. In this article, we utilize the definition of adaptive according to the developmental psychopathology model (see Ellis et al., 2012) as referring to behaviors that augment an individual’s wellbeing, cooperation, and social integration (e.g., Kochenderfer-Ladd, 2004). The idea here is not to propose a ‘new model’ of EI, but to look at the way in which these difference approaches of looking at EI may interact with one another to better predict performance and behavior.

### The PAT Integrated Framework

**FIGURE 1:**
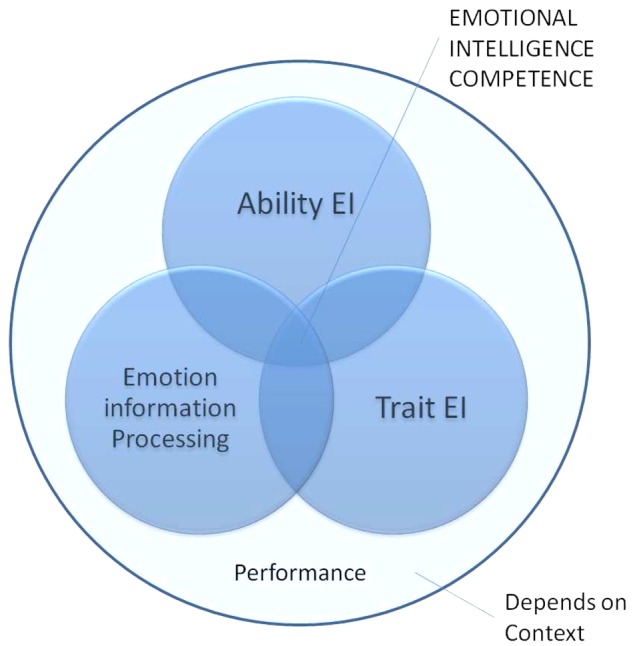
PAT Integrated Framework for the prediction of EI behavior.

Situated within a specific context, the PAT (representing EI**P** – **A**EI – **T**EI) integrated framework includes each of ability EI (AEI), trait EI (TEI), and Emotion Information Processing (EIP). The framework (**Figure 1**) poses the following basic assumptions:

(1) Each EI component is relatively independent.
(2) There is a two-way interaction between each EI component.
  (a) AEI × TEI
  (b) EIP × AEI
  (c) TEI × EIP
(3) *Emotional Intelligence competence* consists of the interaction of all three components
  (a) All three components do not necessarily contribute equally but are always at play.
  (b) The level of EI competence, and thus performance, can be context-dependent.
  (c) Context can also influence the level of contribution of each EI component.

#### Each EI Component Is Relatively Independent

Each component of EI is different and could independently predict behavioral outcomes. Congruent with the literature, each of trait EI, ability EI, and emotion information processing has been previously established as a valuable construct in predicting behavior and performance. Trait EI has long been linked to outcomes related to emotionally intelligent behavior, such as better coping and reduced exam-related stress (Austin et al., 2010), higher levels of leadership (George, 2000; Caruso et al., 2014), workplace flourishing and performance (Di Fabio and Saklofske, 2014; Wan et al., 2014), higher educational achievement (e.g., Bar-On, 2004; Zeidner et al., 2012), and various other physical and/or psychological outcomes (e.g., Palmer et al., 2002; Slaski and Cartwright, 2002; Vesely et al., 2014).

Similarly, the predictive value of ability EI has shown convergence for a wide range of similar outcomes, also showing associations with improved health and wellbeing variables such as stress, coping, teacher efficacy, job satisfaction and performance, social effectiveness and academic achievement (e.g., Brackett et al., 2004; Lyons and Schneider, 2005; Fiori, 2015). Further, and in response to various criticisms, both trait EI and ability EI have repeatedly shown to have incremental validity above and beyond personality and general intelligence on a wide range of outcomes (e.g., Joseph and Newman, 2010; Siegling et al., 2015a; Andrei et al., 2016).

The role of emotion information processing in predicting several emotion-related outcomes is documented in the literature. For instance, research has shown that attention to threatening information, which is particularly developed in high anxiety individuals, may be an advantage when the situation requires high vigilance, but may also impair performance in the case of highly demanding tasks (e.g., Matthews and Dorn, 1995). Results from the broader literature of emotion suggest that high EI individuals may, in principle, benefit from a modulation of emotion information processing (such as either hyphened attention, or inhibition of the processing of emotion information) depending on the situation.

Within the EI literature, previous works have investigated the association between EI and several types of emotion information processing (Gutiérrez-Cobo et al., 2016), such as inspection time (Austin, 2005; Farrelly and Austin, 2007), or attention to emotion information (Fiori and Antonakis, 2012). A recent study (Elfenbein et al., 2017) introduced the regulation of attention toward and away from emotional stimuli as an emotion information processing tasks associated with ability EI and predictive of subjective well-being.

#### There Is a Two-Way Interaction Between Each EI Component

With reference to each of the proposed two-way interactions, we theorize that each component of EI also has the potential to interact with each other. Few studies have assessed both ability EI and trait EI within the same dataset, with some of them theorizing how they may be related to each other. For example, Salguero et al. (2015) examining a sample of female students, found that the relationship between ability EI and symptoms of depression was negative only when individuals were high in perceived EI. The vast majority of studies assessing the impact of both trait EI and ability EI together on the same outcome variables have considered the two perspectives as complementary, both theoretically and within their specific studies (e.g., Mikolajczak, 2009; Foster and Roche, 2014; Gardner et al., 2014) and have not tested the hypothesis that ability EI and trait EI may jointly contribute to emotionally intelligent outcomes. For instance, ability EI and trait EI were shown to contribute independently to performance on a Theory of Mind Task (Ferguson and Austin, 2010) and ability EI and trait EI have shown differential impact on mental health outcomes through diverse roles on coping (Davis and Humphrey, 2012).

Additionally, though no studies of emotion information processing interacting with ability EI or trait EI have been conducted, Austin (2010) showed that ability EI (specifically understanding of emotions) predicted inspection time on an emotion perception task for aspects involving more conscious emotion information processing, raising questions around the means by which these interact at automatic versus voluntary levels. Within the broader literature of cognition and intelligence, aside from seminal theoretical frameworks interpreting the role of information processing in linking personality to performance (e.g., Humphreys and Revelle, 1984), additive effects of personality and speed of processing were found to predict performance on intelligence tests (Bates and Rock, 2004). Interestingly, very little in the literature has been done in conceiving of (emotion) information processing as a variable that may interact with personality and/or ability to produce higher performance. In our framework, the joint contribution of emotion information processing with each of ability EI and trait EI is expected in light of potential beneficial effects of information processing in boosting the effects of cognitive (ability) or personality (trait) individual differences on task performance. For example, within a cognitive task, individuals who are higher on neuroticism tend to look at threatening stimuli more, which may then negatively affect performance. However, individuals who are high on neuroticism, but also good at inhibiting attention to negative stimuli, may lessen this effect.

#### *Emotional Intelligence Competence* Consists of the Interaction of All Three Components

The idea is that EI competence includes contributions from all three components: emotional knowledge and its application (ability EI), processing of emotion information (emotion information processing), and a disposition toward a certain action (trait EI). However, there must not necessarily be equal contribution from each component, as various factors, both individual and contextual, may influence each component, and thus, overall performance. Dependent on the type of performance, joint contribution might be more relevant than independent contributions, such as in the example above where compensation leads to a better outcome than just having personality or information processing alone.

In order to illustrate how the framework with its different assumptions may account for performance, we provide an example of a situation of interpersonal conflict at work in which a customer service representative has to deal with a customer who is unsatisfied with a product. In this example, we identify emotionally adaptive behavior as performing the appropriate actions in order to calm the customer down and end the communication with the customer being less upset than he or she was at the beginning of the interaction. In order for this to happen, one may argue that it would be necessary for the customer service representative to have the knowledge of what types of emotions people (self and others) might feel when they are dissatisfied and understand how this, in turn, would affect their behavior (ability EI). One would also have to recognize one’s own tendency to behave in a certain way given this type of conflict situation (one’s typical response or trait EI), and be able to attend to and inhibit possible maladaptive responses in the moment (e.g., being able to hold back), while also expressing oneself appropriately (e.g., tone and word use) despite possible interfering anger/frustration (emotion information processing). Higher EI competence in some situations may be present with a different ratio of contribution from each EI component, with each having the capacity to affect the other(s). For instance, one may be able to compensate for lack of knowledge about what the other interaction partner feels by applying a habitual response that is usually effective to reduce conflict (e.g., making a joke about the situation). Each of these EI aspects and/or their possible interactions with one another would contribute to how people react in a wide range of situations and are thus included in our approach to predicting emotionally intelligent behavior.

Utilizing variations of the above scenario, **Table 3** provides some examples of how the three EI components may interact to produce more or less adaptive outcomes. One can see how the salience of one or more of the EI components and their interaction could result in these adaptive or maladaptive outcomes (also depending on contextual and personal factors). For instance, if conflict situations are quite common for an individual because he or she works in a customer service complaints department, one might argue that trait EI would present as most salient with the level of emotionally intelligent behavior also being impacted by one’s previous knowledge of the emotional experience (ability EI). Here, the processing of emotion information in the moment (emotion information processing; physiological reactions and focus) is generally habituated. If it is a typical day and the individual is generally a sociable person, has the tendency to try to see things from the perspective of others (high trait EI), and understands how to be effective at problem-solving when others are upset (high ability EI), the likelihood of helping the customer hang up the phone happy is much higher (i.e., emotionally competent behavior). In this case emotion information processing may not have much of an impact.

In another situation, ability EI and emotion information processing may act more strongly – for example, where one could rely on knowledge to express oneself effectively. Congruent with the literature on the impact of declarative knowledge on academic outcomes (e.g., Hailikari et al., 2008; Dunlosky et al., 2013), when given time in which one may prepare, can rely on strategy, and can use one’s knowledge to predict future events, one may perform more effectively. This may be the case for a person who is new to a customer service complaints department but has taken classes about conflict resolution. In this case, the person has little previous experience in the field, but has learned how to deal with problems and how to understand the conflict situations. With the help of some inhibition processes (high emotion information processing), the person could deal more effectively with the complaints of the clients. Hence, various combinations of EI components could result in more or less favorable outcomes.

In summary, one of the main features of the proposed PAT integrated framework (see **Figure 1**) is that it proposes each EI element to predict performance individually as well as when interacting with each of its counterparts. Further, EI components within an interaction can lead to different outcomes (higher or lower) than merely the additive effect(s) of each EI component individually.

Furthermore, the joint contribution of all three EI components characterizes *emotional intelligence competence*. This interaction is called ‘competence’ because it includes and integrates all the components from the literature that have shown to contribute to effective behavior, including declarative and procedural knowledge, past experience, capacity to execute certain tasks, and habitual responses. The salient feature of EI competence *at high levels* is that it ensures the most effective performance by integrating the different components in a way that allows them to compensate for reciprocal shortcomings. Because of this compensatory effect, the predictive ability of the interaction of EI components is seen as *superior* to the predictive ability of the individual contribution of these components, even if one or more components is low. However, whether EI competence (three-way interaction) leads to better or worse performance depends on the EI competence level. This may be either higher or lower as a function of the combination of the three components, thus leading to higher or lower EI performance based on the most adaptive combinations. EI competence best predicts emotionally intelligent performance as it takes all components at stake into consideration and is thus a more accurate representation of the contributors to behavior in emotional situations; however, a two-way interaction, and individual components may also predict performance.

Finally, the framework acknowledges that context may also impact EI competence, and thus performance. EI components may be affected differently by different settings, thus interacting differently and leading to the achievement of more or less emotionally intelligent behavior. For example, in a professional setting, one might have better emotional inhibition skills than in a family setting, where one has habituated to reacting more impulsively given that social desirability may be less demanding.

### The Present Study

The purpose of the current study was to provide initial evidence of the usefulness of the PAT integrated framework in accounting for emotionally laden performance. More specifically, we aimed to test the hypothesis that the integration of the three EI components, or three-way interaction, would predict performance in addition to personality, intelligence, and the direct contributions of each single EI component. Other theoretical models have proposed a distinction of different EI components (e.g., Mikolajczak, 2009), but none to our knowledge have hypothesized the same type of integration of the different EI components in the form of a complex interaction. Furthermore, although the role of emotion-information processing as related to EI has been previously mentioned in the literature (e.g., Roberts et al., 2007) we are not aware of any *empirical* contribution that tested its role in predicting emotion-laden outcomes in interaction with other EI-related components.

Our hypothesis regarding the joint effect of the three EI-components was tested in a task requiring complex mental inferences about another person’s intentions and feelings (a Theory of Mind task). The characterization of Theory of Mind (ToM) refers to the ability to take the perspective of others, specifically to impose their mental states (e.g., beliefs, emotions, desires) on oneself and utilize this information to predict and interpret their behavior (e.g., Premack and Woodruff, 1978; Saxe et al., 2004). This variable is strategically chosen as it is highly relevant to emotionally intelligent behavior, and it has been studied as a separate domain of research from EI.

For instance, a study revealed a positive association between ability and trait EI with performance on tasks measuring social cognitive, but not social perceptual ToM (Ferguson and Austin, 2010). Still under discussion is whether ToM is affected by certain aspects of EI or whether ToM and EI are independent constructs that may interact (Blair, 2002). Evidence suggests that during childhood development ToM influences one’s understanding of emotions, which thus impacts further development of ToM (Meerum Terwogt and Stegge, 2001). The bottom line is that knowledge of emotions, more specifically their emotional understanding, is required in order to develop ToM (e.g., Hughes and Leekam, 2004; Rieffe et al., 2005). Congruent with this, both ToM and EI have been put forth as being important for social interactions (Brackett et al., 2006; Mavroveli et al., 2007; Paal and Bereczkei, 2007).

Further literature examining ToM and EI in the same dataset often recognizes the related nature of these constructs, but also their clear differences. A study looking at these constructs in a population of individuals with Aspergers Syndrome, for example, identifies each as independent explanations for social deficits, utilizing each of these two separate constructs and exploring alternative and additive explanations for their impact on social difficulties (Montgomery et al., 2013). Further, two important studies linking ToM and EI in both children (Qualter et al., 2011) and in adults (Ferguson and Austin, 2010), highlight that these are linked, but distinct constructs, utilizing ToM as an outcome variable and EI as a predictor in both studies. Recent literature has also highlighted the complex nature of ToM and its numerous non-emotional components including those that are cognitive or behavioral (Baker et al., 2014), further supporting the idea that ToM is a broader concept than EI, which includes making inferences on different types of mental, and not only emotional, states of others (see also Ferguson and Austin, 2010).

We set out to look at the impact of all three components of EI, as discussed by our framework, on a ToM task that required identifying the mental and emotional states of individuals depicted only through their eyes. Inferring attitudes and intentions in this way is a behavior that each individual is engaged in on a daily basis, at least any time an interpersonal interaction occurs. Because of its importance in socially adaptive behavior we employed this task in the current research as an example of emotionally intelligent behavior. Regarding the different EI components, we selected only some aspects of each. Concerning the measure of trait EI, we employed the subscale of emotionality because, theoretically, it relates the most to tasks involving emotional cues and making inferences regarding the emotional state of another individual. In addition, this subscale is one of the trait EI dimensions that shows incremental validity on wellbeing-related outcomes (Siegling et al., 2015b) and loads the most into the global trait EI dimension (Laborde et al., 2016). Regarding ability EI, we chose Emotion Understanding as it has shown to load the most strongly onto the latent factor of EI (MacCann et al., 2014). Finally, as for emotion information processing, we employed a task that captures attention to emotional stimuli: the emotional Go/Nogo task (Casey et al., 1997). The capacity to engage attention toward emotional stimuli and disengage from it when needed have been discussed as promising emotional processing underlying the functioning of EI (Fiori, 2009; Fiori and Antonakis, 2012), thus we included them in the current investigation.

## Materials and Methods

### Participants

Participants included 400 undergraduate and graduate students (46% female), with an age range from 17 to 48 (*Mean* = 21.41 and *SD* = 3.27). Participants were recruited from a Swiss University. Participants were required to have fluent knowledge of English as all the tests and questionnaires were in English.

### Measures: Main Variables

#### Demographic Information

Questions included information related to age, sex, grade level, program of study, and English level.

#### Trait EI

The Trait EI Questionnaire-Short Form (TEIQue-SF; Cooper and Petrides, 2010) is a 30-item self-report measure that yields a global trait EI score and four factor composites, including Well-Being, Self-Control, Emotionality, and Sociability. Participants indicate their responses on a 7-point Likert scale (1 = Strongly Disagree, 7 = Strongly Agree). An example of an item is as follows, “Many times, I can’t figure out what emotion I’m feeling.” This measure aims to assess the individual’s self-perceived abilities and behavioral dispositions. The TEIQue emotionality subscale was chosen to represent the trait EI portion of the model as it has shown to be the subscale that loads the most into the global trait EI (Laborde et al., 2016) and, in our sample, it showed the lowest shared variance with personality. According to Petrides (2009) the TEIQue–SF has an internal consistency of 0.88 for global trait EI. The alpha-level for global Trait EI in the current sample was 0.83 and 0.56 for Emotionality.

#### Ability EI

The Situational Test of Emotional Understanding-Short Form (STEU; MacCann, 2006; MacCann and Roberts, 2008) is a 42-item measure that tests the respondents’ knowledge of which emotion is most likely to be felt in a range of situations. It is a performance-based measure of EI that covers 14 emotions in total. Scoring of answers as correct/incorrect is based on Roseman’s (2001) appraisal-based emotion model. An example of an item: “Xavier completes a difficult task on time and under budget. Xavier is most likely to feel? (Pride) The alpha level reported by the authors is 0.67 (MacCann and Roberts, 2008) and it was 0.62 in our sample.”

#### Emotion Information Processing

An emotional Go/Nogo task was utilized as a means of measuring emotion information processing (EIP). This task involves responding as fast as possible to emotional cues–in this case face expressing certain emotions–which corresponds to given criteria, such as faces expressing happiness. These ‘go’ trials are interspersed with trials—the Nogo trials–to which individuals do not have to respond because they do not correspond to the given criterion, such as a neutral face when the criterion is to respond to a happy face. The task yields four types of answers: correct responses to the go trials (or true positive), incorrect responses to the go trials (false positive), correct responses to the nogo trial (true negative) and incorrect responses to the nogo trials (false negative). In the current study we employed the same protocol used in previous studies (e.g., Tottenham et al., 2011), which is characterized by the prevalence of go trials (70%) over Nogo trials (30%). Pictures of neutral faces were always interspersed with emotional faces expressing one of four emotions (happiness, fear, anger, and sadness). There were 12 practice trials followed by 8 blocks of 30 trials each in which the same emotional pair (e.g., happy–neutral) was alternated in three cycles of 10 trial each in which there was a percentage of go trials of either 100, 70, or 30%. Participants responded to 240 trials overall. Stimulus duration was 500 ms with 1000 ms between trials to ensure that participants had enough time to respond. We recorded the overall number of correct answers and errors hits to the go and nogo trials; furthermore, we factor analyzed the four types of answers and identified two main latent factors accounting for 97.7% of the scores, which were retained for further analysis and indicated difficulties in maintaining focalized attention (EIP_DA), and difficulties to inhibit emotional responses (EIP_DI).

#### Reading the Mind in the Eyes Test

The Reading the Mind in the Eyes Test (Revised and online version; Baron-Cohen et al., 2001) assesses an individual’s ability to perceive the mental state of others using an image of only their eyes. It is recognized as a measure of ToM (e.g., Oakley et al., 2016). It consists of 36 gray-scale photos of people taken from magazines. These photos are cropped and rescaled so that only the area around the eyes can be seen. Each photo is surrounded by four mental state terms and the participant is instructed to choose the word that best describes what the person in the photo is thinking or feeling. Participants were instructed to select the most appropriate item and responses were coded as correct or incorrect. The alpha level was 0.63.

### Measures: Control Variables

#### Brief HEXACO Inventory

The Brief HEXACO Inventory is a 24-item questionnaire that assesses the 6 HEXACO model personality dimensions: honesty, emotionality, extraversion, agreeableness, conscientiousness, and openness (BHI, De Vries, 2013). Participants are asked to respond these self-reflective items using a 5-point Likert scale ranging from 1 = strongly disagree to 5 = strongly agree. The alpha reliabilities of the dimensions range between 0.43 and 0.72 (De Vries, 2013). Reliabilities in our sample ranged from 0.36 to 0.57, which may appear low values, but ultimately reflect the few number of items per scale.

#### Verbal Reasoning Test

The Verbal Reasoning test from the Kit of Factor-Referenced Cognitive Tests (Ekstrom et al., 1976) was used to assess a domain of intelligence. This particular cognitive factor is assessed by asking participants to reason with both visual and verbal information in order to draw inferences regarding relationships. Individuals had 8 min to solve 30 problems. Reliability for this sample was 0.65.

### Procedure

The data presented here were part of a larger National Science Foundation Project on the investigation of EI, which has received research ethics approval from the authors’ University ethics committee. Students were recruited from several French-speaking Swiss universities and participated by first filling out questionnaires online and then in a lab session. They gave written consent to participate in the study and received monetary compensation (60 CHF) for a full 3-h session. To participate to this study, students were asked to have a good level of English because all the administered questionnaires, tests, and exercises were in English.

### Statistical Analysis

Descriptive statistics and correlations were calculated including means, standard deviations, and Pearson Correlations using the Statistical Package for Social Sciences version 22 (IBM SPSS Statistics 22; SPSS Inc., Chicago, IL, United States). Multiple regressions analysis using Stata 14 (StataCorp, 2015) were used to test the three-way interaction effects between trait emotional intelligence (TEI), ability emotional intelligence (AEI), and the two indicators of emotion-related information processing task (EIP) on the performance variable (e.g., number of correct answers in the reading the mind in the eyes test). Verbal reasoning, sex, English level, and personality traits were included as control variables.

All independent variables were mean-centered prior to computing their respective product terms, to improve interpretability of the regression coefficients and reduce collinearity between the 3-way interaction predictors and the main effects (Schielzeth, 2010). Four interaction terms were included in the analysis—AEI X TEI, AEI X EIP, TEI X EIP, AEI X TEI X EIP. As EIP was assessed by two indicators, difficulties in maintaining focalized attention (DA), and difficulties to inhibit emotional responses (DI), two separate regressions were run to test both 3-way interactions as predictors of performance (the Reading the Mind in the Eyes test).

## Results

**Table 1** shows Pearson correlations for independent, dependent, and control variables. Reading the Mind in the Eyes (RME) was correlated significantly with all three EI components, except with EIP-DI, and mostly strongly with EIP-DA (*r* = -0.36); their correlations are quite modest, supporting the idea that EI and ToM are related but distinct constructs. In congruence with previous research, Ability EI and Trait EI showed a low but significant correlation (*r* = 0.12; e.g., Vesely et al., 2014). Both indicators of EIP were correlated with Ability EI but not with Trait EI. Of the control variables, agreeableness and emotionality were not correlated significantly with any of the independent or dependent variables. Honesty, extraversion, conscientiousness, and openness to experience were correlated significantly with one or more of the independent variables. The intelligence proxy variable, verbal reasoning, had a significant, moderate correlation with Ability EI (*r* = 0.35), EIP-DA (*r* = -0.25), EIP-DI (*r* = -0.16), and RME (*r* = 0.35). Gender was also significantly correlated to Ability EI (*r* = 0.15) and RME (*r* = 0.20) and English level significantly correlated to Ability EI, EIP-DA, and RME. Only honesty and openness from the personality traits were entered as control variables in the final regressions due to the lack of association of the other personality traits with the dependant variable.

**Table 1:** Means, standard deviations, and correlations.

	*n*	Mean	*SD*	STEU	TEIQue	EIP-DA	EIP-DI	RME	H	Em	Ex	A	C	O	VR	Gender	EL
STEU	400	15.57	3.50	1													
TEIQue	400	4.65	0.75	0.12*	1												
EIP-DA	394	0.06	0.03	–0.33***	–0.06	1											
EIP-DI	394	0.09	0.06	–0.12*	–0.05	–0.00	1										
RME	329	26.24	4.20	0.35***	0.14*	–0.36***	–0.10	1									
H	400	14.60	2.77	0.13*	0.14**	–0.09	–0.06	0.15**	1								
Em	400	11.36	3.17	–0.04	0.02	0.06	–0.01	0.07	0.12*	1							
Ex	400	15.29	2.34	0.16**	0.22***	–0.14**	–0.14**	0.10	0.10	–0.03	1						
A	400	11.50	2.54	0.04	0.10	0.03	–0.05	–0.09	0.17**	–0.08	0.01	1					
C	400	13.95	2.83	–0.03	0.18***	0.02	–0.03	–0.00	0.16**	–0.04	0.16**	0.02	1				
O	400	15.09	2.51	0.04	0.19***	–0.04	0.00	–0.17**	–0.09	–0.09	0.13*	–0.03	0.01	1			
VR	400	12.33	3.79	0.35**	0.07	–0.25***	–0.16**	0.35***	0.16**	–0.03	0.03	0.07	–0.05	0.13*	1		
Gender	400	0.46	0.50	0.15**	0.07	–0.09	–0.00	0.20***	0.22***	0.43***	0.11*	–0.07	–0.01	0.07	–0.02	1	
EL	400	3.51	0.66	0.19***	0.08	–0.16**	0.06	0.22***	0.03	–0.05	0.05	–0.05	–0.08	0.09	–0.18***	0.06	1

*n, total sample size; STEU, Situational Test of Emotional Understanding; TEI, emotionality subscale of Trait Emotional Intelligence Questionnaire -Short Form; EIP-DA, difficulties in maintaining focalized attention in the emotional Go/Nogo task; EIP-DI, difficulties to inhibit emotional responses in the emotional Go/Nogo task; RME, Reading the Mind in the Eyes; H, Honesty; EM, Emotionality; Ex, Extraversion; A, Agreeableness; C, Conscientiousness; O, Openness; VR, verbal reasoning; EL, english level.*

*^∗^p < 0.05, ^∗∗^p < 0.01, ^∗∗∗^p < 0.001.*

The results of the multiple regression analysis to test the 3-way interaction effect of Ability EI, Trait EI, and *EIP-DA* on the reading the mind in the eyes task showed that the 3-way interaction term was significant (see **Table 2**). All the 2-way interactions and the main effect of Trait EI were not significant. On the other hand, the main effects of Ability EI and difficulties to inhibit emotional responses were significant. Verbal reasoning, gender and English level predicted all three significantly the score on the reading the mind in the eyes task. *R*^2^ was 31,49%.

**Table 2:** Summary of multiple regression analysis for variables predicting reading the mind in the eyes (*N* = 323).

Variable	*B*	*SE B*	β	*t*	*p*
**DV: RME**					
AEI	0.17	0.06	0.14	2.60	0.010
TEI	0.31	0.29	0.05	1.07	0.286
EIP-DA	–0.65	0.25	–0.16	–2.59	0.010
AEI × TEI	–0.14	0.08	–0.09	–1.81	0.071
AEI × EIP-DA	–0.03	0.05	–0.04	–0.60	0.551
TEI × EIP-DA	0.39	0.31	0.07	1.26	0.207
AEI × TEI × EIP-DA	–0.19	0.08	–0.15	–2.37	0.018
AEI	0.23	0.07	0.19	3.41	0.001
TEI	0.45	0.29	0.08	1.53	0.128
EIP-DI	–0.20	0.21	–0.05	–0.93	0.351
AEI × TEI	–0.15	0.08	–0.09	–1.88	0.061
AEI × EIP-DI	–0.02	0.06	–0.02	–0.34	0.736
TEI × EIP-DI	–0.03	0.28	–0.01	–0.12	0.904
AEI × TEI × EIP-DI	–0.12	0.08	–0.08	–1.53	0.127

*Results of block 1 with only control variables are not reported. AEI, ability emotional intelligence; TEI, trait emotional intelligence; EIP-DA, emotional information processing—difficulties in maintaining focalized attention; EIP-DI, emotional information processing—difficulties to inhibit emotional responses.*

The results of multiple regression analysis to test the 3-way interaction effect of Ability EI, Trait EI, and *EIP-DI* on the reading the mind in the eyes task showed that the 3-way interaction term was non-significant as well as all the 2-way interaction and the main effects of Trait EI and EIP-DI. Only Ability EI had a significant main effect on the outcome.

**FIGURE 2:**
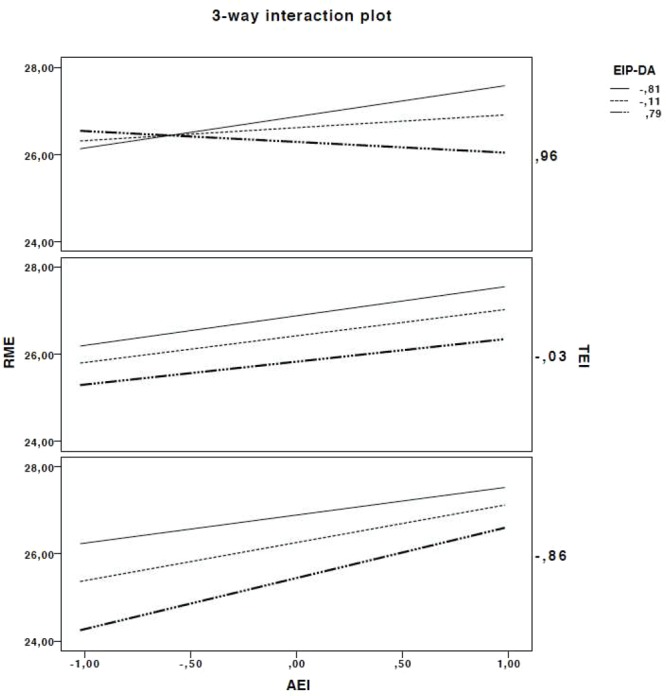
Three-way interaction plot of AEI, TEI, and EIP-DA RME (*N* = 324). AEI, ability emotional intelligence; TEI, trait emotional intelligence; EIP-DA, emotional information processing—difficulties in maintaining focalized attention; RME, Reading the Mind in the Eyes.

**Figure 2** shows the three-way interaction plot indicating the relationships between Ability EI, Trait EI, and Emotion Information Processing-difficulties to maintain focalized attention responses (EIP-DA) and their combined impact on RME. The top plot shows that when Ability EI and Trait EI are both high, low percentage of errors in EIP-DA result in better performance on RME. When Trait EI is high and Ability EI is low, Emotion Information Processing does not impact the scores on RME. Referring to the middle graph, when the Trait EI score is average, Emotion Information Processing impacts performance regardless of the level of Ability EI. The bottom plot shows that when Trait EI and Ability EI are low, Emotion Information Processing seems to impact scores on RME: low scores on AEI, low scores on TEI, but a high EIP, result in better performance. When Trait EI is low and Ability EI is high, Emotion Information Processing does appear to improve only slightly the performance on RME.

## Discussion

The present study proposes a new framework integrating three approaches to conceptualizing the best predictor of emotionally intelligent behavior: trait EI, ability EI, and emotion information processing. It then empirically tests the framework’s ability to predict adaptive performance by means of an interaction of the three EI components using an emotionally laden ToM task in a sample of university students. Results reveal the proposed model to be a good fit for the data. The multiple regression analysis indicated that when all EI components as well as interactions are included in the model, performance can be predicted by a three-way interaction between Ability EI, Trait EI, and Emotion Information Processing. The three-way interaction, what we have characterized as EI competence, was able to predict variance in ToM (measured by RME) above and beyond the role of each EI individually and when controlling for personality and intelligence. In addition, the three-way interaction predicted performance on top of the ability and trait EI interaction, showing that the inclusion of emotion information processing as a complementary EI component may help to better predict emotion-laden performance.

Looking at the more specific relationships between Ability EI, Trait EI, and Emotion Information Processing, different combinations and levels of each EI component seem to yield different outcomes in ToM. It seems that high Emotion Information Processing may boost performance when either the individual has both low Trait EI and Ability EI, or when the individual has high Trait EI and high Ability EI. In other words, it seems that Emotion Information Processing, in particular being capable of maintaining focalized attention, boosts the effect of trait and ability EI and exerts a compensatory effect for deficiencies in Trait and Ability EI. In the specific case of the Reading the Mind in the Eyes task performance, our results suggest that focalizing attention on the emotional cues of the task stimuli (such as the shape of the eyes or the direction of the eyesight) helped to score higher when individuals were falling short on the ability to understand emotions (Ability EI) and the habitual responses to emotional situations (Trait EI).

Overall, results highlight the important role of the interaction of the different EI components in predicting higher performance. Indeed, such components are often investigated individually, but omitting the measurement of the other components may provide only a limited representation of emotionally intelligent behavior. One of the most important advantages of investigating the join contribution of Ability EI, Trait EI, and Emotion Information Processing is that each component may compensate for the lack of the other components, ultimately leading to better performance. The predictive ability of the three-way interaction (EI competence) is superior to the predictive ability of the additive effect of each EI component individually as the interaction of EI components can change the outcomes for better or for worse.

A concrete example may be that, in the measurement of racing speed, engaging in a certain amount of weight training and cardio together would improve race times more or less than just the additive effect of either weight training and cardio individually (i.e., individually they would remove 3 s plus 4 s from one’s race time vs. in the case of an interaction – they would remove more than 7 s – perhaps because, combined, they result in greater gains due to the way in which muscles recover when doing cardio). It could also mean that neither has an effect on speed unless both contribute. The more EI components are accounted for, the more accurate the representation obtained of emotionally intelligent behavior can be. In the PAT integrated framework, high EI competence can be achieved when all three EI components interact with each other at optimal levels, resulting in the best possible performance.

The noted race example as well as those examples presented in **Table 3**, each showing how EI components may interact and thus lead to more or less adaptive outcomes, are congruent with the majority of literature on competence and general learning indicating that different types of knowledge, characteristics, and processes [e.g., declarative/procedural knowledge; implicit/explicit processes (in this case emotional knowledge/processes)] can work together to be effective at performing a range of tasks competently (Anderson, 1983; Bellier, 2004). Additionally, they fit with examples from the literature in which individuals are taught skills in one domain to compensate for deficiencies in others within emotionally laden situations. For example, this has been demonstrated in individuals engaged in cognitive behavioral therapy for anxiety, where cognitive reappraisal is utilized to reduce fear responses to certain stimuli (Beck, 2011). Further, and more specific to EI, a variety of outcome variables have been studied measuring both Ability EI and Trait EI within the same sample, indicating the importance of both, but in different ways. For example, Ability EI and Trait EI showed differential impact on mental health outcomes through diverse roles on coping. Specifically, Ability EI was described as driving the selection of coping strategies, whereas Trait EI influenced coping efficacy, in other words their later implementation (Davis and Humphrey, 2012). Results such as these are relevant to the current study as they show further evidence of various aspects of EI contributing differently to important outcomes.

**Table 3:** Hypothetical examples of EI interactions.

Individual Factors (expectations; typical or atypical; experiences; practice)	TEI	AEI	EIP	Outcome and explanation
This happens daily; you have been working there for years	**High** Your habitual emotional response, (which is adaptive) is ready and comes relatively automatically	**Average to High** You have the emotional knowledge of how to deal with this situation and experience doing so	**Low** Your brain and body do not effectively process the emotion-related information **High** Your brain and body can effectively process the emotion-related information	**More adaptive outcome (both)** EIP does not seem to be utilized as much as TEI and AEI (e.g., inhibition of a negative reaction is not required because your habitual reaction is adaptive)
It is your first day on the job; you rarely deal with conflict in general	**Low** You resort to a habitual emotional response that is inappropriate in professional circumstances (e.g., invalidating the customer’s point of view)	**Low** You have never worked in customer service and have poor knowledge of emotion management	**Low** Your brain and body do not effectively process the emotion-related information **High** Your brain and body can effectively process the emotion-related information	**Less adaptive outcome (both)** Even inhibition of a negative habitual emotional response could not help as you do not have a knowledge base to draw from
It is your first day on the job; you have taken classes on conflict resolution; you expect conflict	**Low** You habitual response is inappropriate in a professional circumstance (e.g., making a joke)^∗^	**High** Your theoretical learning provided you with a good emotional understanding of the customer’s perspective	**High** Your brain and body can effectively process the emotion-related information and **Low** Your brain and body do not effectively process the emotion-related information	**More adaptive outcome** You use AEI and EIP together to override TEI (i.e., you inhibit your tendency to tell a joke and express an adaptive phrase according to your understanding) **Less adaptive outcome** Your AEI is overridden by low EIP and low TEI [e.g., history of negative emotional experiences take over and you impulsively resort to your habitual response (make an inappropriate joke)]
You have dealt a lot with conflict in a previous position, but you do not seem to have consistent outcomes	**High** You are self-confident and pride yourself in identifying the emotional responses of your clients	**Low** Though you think you know - you do not have a good understanding of emotion management and you misunderstand many emotionally laden situations	**High** Your brain and body can effectively process the emotion-related information and **Low** Your brain and body do not effectively process the emotion-related information	**More adaptive outcome** Though AEI is low, your high TEI and high EIP can compensate (e.g., your attention to the client’s emotions validates their experience despite your lack of understanding) **Less adaptive Outcome** Your lack of attention to the client’s emotions and your lack of understanding do not complement your high TEI

*Not all possible combinations from the model are exemplified here; just some salient examples. ^∗^This is assuming that the course did not improve TEI.*

*Context: You are involved in a conflict situation at work where you hold a customer service position. The customer is upset with the product and is yelling at you.*

### Limitations

We believe we have provided initial evidence supporting the utility of using an integrated approach in investigating predictors of emotionally intelligent performance; nevertheless our results warrant replication because of the following limitations. The use of short-forms of measures (e.g., TEIQue-SF; HEXACO) as well as the general use of self-report questionnaires have acknowledged limitations, such as some low reliability values, especially for the emotionality subscale of the TEIQue and for the HEXACO personality traits due to the few items included in the scale, together with common method bias, vulnerability to social desirability, and ecological validity (Grubb and McDaniel, 2007; Roberts et al., 2007). In our study we found a low reliability for the emotionality subscale of the trait EI questionnaire, for this reason results should be taken with a note of caution.

This study also utilized only one subscale (or aspect) of each EI component (i.e., emotion understanding for Ability EI; emotionality for Trait EI; emotional Go/Nogo for Emotion Information Processing). Though these were chosen strategically as (a) these subscales are the most relevant in relation to ToM and other emotionally intelligent outcomes and (b) the emotionality subscale from the TEIQue has shown the lowest shared variance with personality in our sample, replication with more complete measures as well as with other outcome variables and additional experimental conditions (such as those measuring stress inoculation), could provide a more comprehensive understanding of the PAT integrated framework.

Replacing each measure of EI with different measures utilizing different theoretical models (such as the Genos 7-factor model rather than the TEIQue and the GERT/STEM in addition to the STEU) could also add to future consideration. Further, novel measures of EIP that measure individual differences need to be developed. In our study we employed a typical emotion information processing task employed in the emotion and cognition literature, however, the emotional Go/Nogo is not generally employed as a stable individual difference measure in attentional processes. New measures that capture both attentional processes and that can reliably differentiate individuals still need to be introduced in the EI literature. Additionally, given the possibility that use of an undergraduate student sample resulted in higher scores on measures of daily functioning and well-being (such as EI and IQ) than found in the general population, a different sample should be used to assess the model once again.

### Implications and Future Directions

Among the most important implications of our study, the understanding of which aspect of EI is ‘deficient’ may allow us to identify where to provide intervention. Previous EI training programs have shown to be effective in improving EI as well as in increasing performance outcomes in different fields (Nelis et al., 2009, 2011; Pool and Qualter, 2012). For example, Di Fabio and Kenny (2011) used a short training program focusing on Trait EI improvement where post-program results indicated that students had less difficulty related to career decision-making. Further, in the domains of psychology and education, results of EI training (programs utilizing Ability EI and Trait EI models) have indicated not only that EI improves post-program compared to controls at follow-up, but also that stress decreases alongside increases in adaptive coping and other outcome variables like teacher efficacy (Brackett et al., 2011; Vesely-Maillefer, 2015). Though Emotion Information Processing is a new domain in EI research, studies conducted on inhibition and attention processes in clinical psychology have shown that deficits in these domains can be improved through various interventions (e.g., Shafir, 2015; Scott and Weems, 2017). The positive impact of EI training coincidental with the knowledge that each EI attribute may contribute to performance outcomes individually and interactively, supports the need for further studies combining training aimed at improving Ability EI, Trait EI, and Emotion Information Processing.

In sum, we have provided the first theoretical and empirical account for how the different components of EI—which traditionally have been investigated as different approaches in studying EI—may jointly predict emotionally intelligent behavior. The integration of these different components of EI into a more comprehensive framework allow us not only to bridge the gap between the terminology and conceptual inconsistencies within the literature, but it also makes both empirical and theoretical sense based on previous research. The PAT framework proposed has several important features. It provides a rationale as to why each EI component contributes to emotionally intelligent behavior individually and jointly. It shows how within the interaction of all three EI components (in the current framework conceptualized as EI competence), EI components may compensate for each other in yielding the most adaptive emotional outcomes. Further, it indicates that EI competence at all levels is the best predictor of EI-related performance. It clarifies terminology and conceptual inconsistencies within the literature by making a more clear distinction between predictors and outcomes. Ultimately it also allows for a more inclusive understanding of the way in which EI in general may explain variability in these outcomes. These results provide initial support for exploring further the relationships between different components of EI and to develop comprehensive models for predicting and improving performance in emotionally laden situations.

## Ethics Statement

All subjects gave written informed consent to participate in the study. The study protocol was approved by the Ethics Committee of the Faculty of Business and Economics of the University of Lausanne.

## Author Contributions

AV and MF article conceptualization and write-up. SU article conceptualization, write-up, and data analysis.

## Conflict of Interest Statement

The authors declare that the research was conducted in the absence of any commercial or financial relationships that could be construed as a potential conflict of interest.
